# Local ancestry in Apoe3: Friend or Foe?

**DOI:** 10.1002/alz.091750

**Published:** 2025-01-03

**Authors:** Sofia Moura, Katrina Celis, Luciana Bertholim Nasciben, Farid Rajabli, Joe Rivero, Kara L. Hamilton‐Nelson, Patrice L. Whitehead, Marla Gearing, David A. Bennett, Margaret E Flanagan, Sandra Weintraub, Changiz Geula, William K. Scott, David A. Davis, Regina T Vontell, Theresa Schuck, Derek M. Dykxhoorn, Margaret A. Pericak‐Vance, Anthony J. Griswold, Juan I Young, Jeffery M. Vance

**Affiliations:** ^1^ John P. Hussman Institute for Human Genomics, Miller School of Medicine, Miami, FL USA; ^2^ John P. Hussman Institute for Human Genomics, University of Miami Miller School of Medicine, Miami, FL USA; ^3^ John P. Hussman Institute for Human Genomics, University of Miami Miller School of Medicine, Miami, FL, USA, Miami, FL USA; ^4^ Emory University, Atlanta, GA USA; ^5^ Department of Neurological Sciences, Rush Medical College, Chicago, IL USA; ^6^ Northwestern University Feinberg School of Medicine, Chicago, IL USA; ^7^ University of Miami Miller School of Medicine, Miami, FL USA; ^8^ University of Pennsylvania, Philadelphia, PA USA; ^9^ John P. Hussman Institute for Human Genomics, Miami, FL USA

## Abstract

**Background:**

Non‐Hispanic White *APOE4* carriers have a higher risk of developing AD compared to African American *APOE4* carriers. The local ancestry (LA) surrounding the *APOE* region was previously shown to be the primary factor in this risk difference. *APOE4* carriers of European LA (ELA) have been found to have higher *APOE4* expression and chromatin accessibility compared to African LA (ALA). We sought to investigate whether the LA around *APOE3* has the same effect between ancestries. Differences between alleles could provide insight into ancestry‐specific regulatory areas controlling the *APOE4* expression.

**Method:**

We identified AD autopsy samples by GSA, all homozygous for *APOE3* and LA from 4 ADRC brain banks: Miami, Emory, Duke, and Rush. We performed single nuclei RNA sequencing (snRNA‐seq) on eight frozen frontal cortex (B9 area) samples using 10x Genomics.

**Result:**

We performed snRNA‐seq in a total of 51,462 nuclei from eight brains (4 ELA and 4 ALA). We identified 35 distinct cell clusters at a resolution of 0.6. The proportion of cells per cluster between ELA and ALA samples was similar for all clusters, except for cluster 32 (Excitatory Neurons) which had a greater than 2‐fold difference in ALA. Our data shows that *APOE3* carriers with ELA have a significantly higher *APOE* expression in Excitatory Neurons (cluster 7) than those of ALA and contrary to our previous observations in *APOE4* carriers (Griswold, A. *et al*, (2021)), *APOE3* carriers of ALA express higher *APOE* in astrocytes (cluster 2) and Microglia (cluster 6). However, overall, comparison of *APOE3* vs *APOE4* carriers in this study demonstrated that *APOE3* alleles have significantly lower gene expression than *APOE4* carriers of the same LA.

**Conclusion:**

Our preliminary data suggest that the LA surrounding *APOE3* is associated with different effects on *APOE* expression compared to *APOE4* allele. Further, within the same LA, we observed that, overall, the different cell types express less *APOE* in *APOE3* carriers compared to *APOE4* carriers. Altogether, this may provide additional insight into the regulatory mechanisms affecting *APOE4* expression.

